# Balancing the scale

**DOI:** 10.1002/jeab.792

**Published:** 2022-08-18

**Authors:** Kathryn L. Kalafut, David M. Freestone

**Affiliations:** ^1^ The Chicago School of Professional Psychology; ^2^ Habit Technology Cincinnati

**Keywords:** applied, animal behavior, collaboration, experimental analysis, research

## Abstract

Traditional discussions involving ‘basic’ and ‘applied’ behavioral research often focus on the differences, or gaps, between these areas. They take place in different environments, use different methods, ask different questions, and have different objectives. Applied animal behavior is no exception. Focusing on the differences in these areas is to the detriment of a cohesive and complete understanding of animal behavior. This paper instead focuses on the similarities between these two sides, and presents them as a matter of scale. A series of real‐life examples experienced by the authors is used to highlight how the skills and knowledge of both the applied and the basic sides are valuable and necessary to not only further both fields independently, but to develop a comprehensive understanding of animal behavior.

Discussions of the gap between basic (or experimental) research and applied practice are not new (see Lit & Mace, 2015, for an overview). The area of applied animal behavior is no exception. Having served as professors in departments emphasizing behavior and learning in both research and practice, the authors have witnessed this divide first hand. Students focusing on applied practice often state that the experimental side of behavioral science “does not apply to them”, or “does not impact their practice”. Like many before us (see Sidman, 2011), we believe that work in applied practice can benefit substantially from experience in, or at least an appreciation for, basic research. Similarly, the students of the experimental side are often very interested in seeing their research and methods have a larger impact than ‘knowledge for the sake of knowledge’. It has been our experience that most students of basic research would jump at the chance to share their results, methods, and approaches with those who work in applied practice if it meant having a broader impact or better applied results.

But how does one begin to reconnect, or close, the research–practice loop? Recent attempts to bridge this divide have focused on highlighting translational research, or research that focuses on how basic principles of behavior may be utilized and generalized into everyday problems (Mace & Critchfield, 2010). Though this approach is a valuable one, it suggests a third type of research is necessary to bridge this gap, which can seem daunting. The goal of this paper is to provide an alternative perspective to begin to bridge the gap between basic and applied research. Our approach suggests that basic and applied research are extremes on a spectrum, and the differences between these areas can be viewed in terms of scale. While this idea of a spectrum is not novel (Kyonka & Subramaniam, 2018), by presenting this as a difference in scale we hope that readers will (potentially more clearly) see how the skills and approaches brought by basic and applied areas 1) not only complement one another, but are similar in nature and different in scale, 2) can be used to create more effective practice, and 3) create a deeper understanding of behavior. This perspective aims to demonstrate how valuable this kind of approach can be for those willing to seek it out. We will close with suggestions as to how students, practitioners, and researchers can begin to close this loop.

The four aspects of scale that will be discussed are 1) the number of subjects, 2) the number of measures, 3) the manner of data collection and 4) the manner of data analysis. Our goal is to show the value of closing the research–practice loop by sharing personal experiences from both the laboratory (conducting basic research) as well as applied animal behavior settings (addressing real‐world questions/issues). To start, let's take a look at two scenarios, one from a basic setting, and another from the applied.

A basic researcher sits at their computer, viewing a mirror image of what their research subject, a monkey, sees on the screen in front of them. The monkey has been trained to visually scan the stimuli that appear on the screen, and make a choice (indicated by touching the screen). This choice may lead to a sip of juice, or it may lead to no juice. The experimental protocol, species of the research subject, apparatus, measures collected, and environment have all been carefully chosen to study a specific aspect of decision making. Across town at the zoo, a monkey and the rest of its troop are being shifted from their sleeping area to their outdoor habitat. This individual monkey begins to climb on some of the aerial structures available, and alters its roughness of play as it engages with different members of its troop.

In both of these situations a monkey is making decisions. Both situations are of interest, and the individuals tasked with understanding these behaviors will approach these situations differently. Each approach is useful and fruitful given each environment and the questions being asked. The basic researcher will (hopefully) be able to say something about decision making under these experimental conditions. “These experimental conditions” includes a single monkey, in isolation, and in a very controlled environment. If another monkey were to be introduced into this situation, the results observed from a single monkey would undoubtedly change. The presence of another monkey influences the behavior of the first (and if you add a third, the findings from two may also not hold). This is the first issue of scale between basic and applied work—the number of individuals typically under study. In the basic laboratory, a rat/mouse/pigeon/monkey is typically placed in a chamber/apparatus where inputs and outputs are continuously recorded across trials, for many experimental sessions. In many applied settings, we may be dealing with numerous individuals in the same space, and need to account for the fact that the behavior of an individual may be altered by the presence, absence, or behavior of other individuals, sometimes of a variety of species.

Moving from a single individual to multiple is necessary to truly understand behavior in applied settings. There is no doubt that the basic behavioral principles identified using a single subject will hold when looking at a group of individuals, but the environment is inherently more complex, and requires an understanding of the multiple individuals in the group (both as individuals and species) as well as more complex data collection and analysis. Neither the applied nor the basic side is currently equipped to do this well on their own. To illuminate this point, let's take a closer look at the basic environment in the example above. In this environment, the researcher is looking at an individual monkey's behavior, but they may be looking at a variety of behaviors continuously. The apparatus used in this kind of experiment allows for all inputs (responses from the monkey) and outputs (stimuli presented to the monkey) to be recorded in real time. The inputs may include physical touches to the screen, eye tracking data (where the monkey is looking), how long the monkey licked the juice spout, when they lick the juice spout, etc. From these measures, countless others can be calculated; interresponse times, latency of responding, and so on. This allows the basic researcher to paint a picture of what is happening during each moment of an experimental session through the recorded data. The recorded data coming out of a single experimental session may be hundreds or thousands of recorded instances of inputs and outputs (depending on the experiment, the individual subject, the length of the session, etc.).

Let's juxtapose this with an applied example. A group of penguins at a facility have bumblefoot, or swelling of their feet. The veterinarian staff thinks in addition to medical attention, a behavioral change, increasing swimming time, may help alleviate symptoms. How do you increase the time spent swimming for over 30 penguins? Before you can begin to address that question, you first need to identify how you will measure swimming, and how you will collect these data. Collecting behavioral data over time is essential for the understanding the well‐being of animals under human care (for livestock, zoo and animal shelter examples, see Li et al., 2020; Stephen & Ledger, 2005; Watters et al., 2009, respectively). To understand how much the penguins are swimming, you may decide that having volunteers or staff collect data on swimming for individual animals is the best way to get started. While this may give you an idea of overall swim time, this is not a long‐term solution to see potential changes in swim time. Collecting these data requires time, resources, and staff familiar with data collection methods and analysis, something that has been a limiting factor for animal care institutions, particularly zoos, aquariums, and animal shelters, for some time (Loh et al., 2018; Stoinski et al., 1998). Still, the most common path for these institutions is to find and train observers, who then need to carve out the time necessary to collect data by hand.

This highlights another matter of scale; the number of measures or data being collected. Basic researchers have the luxury of equipment specifically created for continuous, automatic, and dedicated data collection on a variety of measures for their specific research species. Applied settings tend to focus on relatively few measures, and discontinuous measurement systems to collect data. This is necessary given the lack of resources and time in these environments. While *more* measures are not always beneficial, it should be a goal of applied research to collect all the data necessary to paint a full picture of behavior (this could include more measures, and/or more continuous data collection methods). Time and ability do not need to be the factors that limit the number of measures or the amount of data collected. As noted, basic researchers work daily with their research devices, and need to know how to fix and maintain their equipment. This gives them an advantage in terms of understanding how technology can be used to collect behavioral measures, but will not generalize completely to an applied setting. To extend the technology used in the laboratory for automatic data collection, the basic researcher needs help from the applied practitioner. The applied practitioner usually has specific knowledge of the animals they work with, both as a species and as individuals. While they may have certain questions or issues of interest, how to collect data to address them may not be immediately clear (or available given the limitations described above). The knowledge and expertise from both sides is necessary to begin to tackle the gap in the number of measures, and methods used for data collection.

To go back to the penguin example, this was a real question/problem presented to the authors. The solution that was developed was to use radio‐frequency identification (RFID) technology to track the location of the penguins on exhibit and determine when they entered the water (Kalafut & Kinley, 2020). The researchers strategically placed antennas around the penguins' habitat that sent information indicating who (which tag) was detected where (which antenna) and when (the date and time) a tag (placed on the penguin's wing‐band) was in proximity to the antenna. This system collects who, where, and when, continuously and automatically. These data can not only be used to see which penguins are swimming and when, but it can be used over time to see how changes in the environment (water temperature, air temperature, breeding season, number of guests at habitat, enrichment items, etc.) alter the penguins' behavior. For this project to be successful, we needed the expertise of the animal care staff who understand the penguins as a species (species‐typical behaviors, physical capabilities, etc.), as individuals (specific behaviors or issues seen with individual penguins), as well as the behavioral goals and objectives set by the people who know these animals best. We needed to utilize our skills as basic researchers to recognize how technology could be used to create automatic systems of reliable and valid behavioral data collection. More data on a variety of indices should be used when available to enhance our understanding of animal behavior and welfare (Hill & Broom, 2009). But with changes in the kind of data being collected, changes in the kind of analyses must also follow.

Collecting data by hand provides sparse data on a few measures, and allows for generic visualizations to be created with minimal computing power or background in data analysis. A commonly recommended visualization for these hand‐collected data on animal behavior is an activity budget (Wark et al., 2019; Watters et al., 2009), which shows the percentage of time an animal allocated to certain behaviors over a period of time. An activity budget is valuable for a broad overview of the behaviors being observed, but does not provide insights into how to go about behavior change. It cannot tell you anything about *when* these behaviors occur. Do they occur at a specific time of day? Are they seasonal? Do they occur more or less often at times when the data collectors (usually present only during specific time periods) are present? While this generic visualization and others like it have been necessary given the reality of lack of time and resources in many applied settings, they are indeed limiting. The introduction of the RFID system allowed for continuous and automatic behavioral data collection.

These data can be used by both researchers and practitioners for a variety of reasons. Most importantly, it can allow for data‐based decisions to be made regarding the care and best practices for these penguins in real time. But this is only possible once these data have been cleaned, analyzed, and visualized. This solution to collecting better data is followed by another issue of scale, the method of analysis for these behavioral data.

Two further scenarios will be presented to further highlight the importance of methods of data collection and the preparation/analyses necessary to make these data useful. The first scenario starts with a discussion with a Master's student who, having spent quite a bit of time working at a cat shelter, had developed many questions regarding shelter cat welfare. When space is at a premium in a crowded animal shelter, is a double‐sided cage for a cat worth the cost in space for increases in welfare? To answer this question, she wanted information on where the cats were spending time when they had access to either a single or double compartment. To collect these data, again, RFID antennas were strategically placed in locations that would pick up the tag (placed on the cat's collar) and deliver these data continuously and automatically (Barnes et al., manuscript in preparation). But data are only as useful as what one can do with them. This seems like a very generic statement, but it is one worth considering. Devices that automatically collect data don't typically deliver them in a neat package. In this case, the RFID data were delivered as a long list of detections that included the antenna, the tag number, the date and the time. These data are akin to those received in the basic laboratory where the various inputs and outputs are collected continuously with the timestamp of when they occurred. In both instances the researchers are left with a ‘laundry list’ of events that then need to be cleaned, sorted, organized, analyzed, and visualized. This is a level of data analysis that basic researchers are used to. It is one of the necessities of conducting their research. This is not usually the case with applied practitioners. In the case of the shelter‐cat welfare research, these data were able to be analyzed and visualized while the study was taking place, and the student worked through this ‘big data’ approach to welfare by learning the tools and techniques necessary for this kind of data analysis. In the previous example of using the RFID data for real‐time analysis of penguin swimming behavior, additional measures needed to be implemented that allowed not only for the data to be collected automatically, but for the data to be cleaned, analyzed and visualized continuously and in real‐time. This kind of software infrastructure goes beyond simple data analysis, but is necessary for timely data‐based decisions to be made based on current behavior. This is another area where closing the research‐practice loop would benefit applied practice, but can only be done fully through clear communication and collaboration among the two areas, and even in areas outside of behavioral science (software engineering, etc.).

The second scenario began with an off‐hand comment made by a friend and veterinarian. She said, “if only we could get cats to just drink more water, maybe their kidneys would function better.” This conversation led us directly to our current research question: Does an increase in water consumption enhance kidney function in domestic and exotic cats? This provides a good example of a current research question posed by applied practitioners, and where an understanding and appreciation of basic research complements the research in a complete way. In order to determine how increased water consumption alters kidney function, from a behavioral perspective, you need to be able to record the amount of drinking a cat is doing. Most basic researchers are familiar with a device known, aptly, as a lickometer. It is a device used to measure the number of licks to a water spout. This is a piece of basic research equipment that is fairly standard to a basic laboratory for use with rats, mice, and monkeys (albeit usually quite expensive). A basic researcher may have insights into its maintenance and function. While there are plenty of resources on how to build this device on your own (Godynyuk et al., 2019; Raymond et al., 2018) for use with rodents, careful adjustments are required for use with cats (or presumably, any other species for which you might want to measure licks). In addition to the equipment that might be of value for this research, drinking behavior is something that has been studied extensively in the basic literature.

In the laboratory, researchers have been able to create environments that produce polydipsia, or excessive drinking, by manipulating schedules of reinforcement (Falk, 1966). While excessive drinking is not the desired outcome, these basic findings may shed light on how to increase drinking behavior simply through different schedules of reinforcement. But, again, the basic research findings come from a controlled environment with a single animal under strict experimental control. Will these results extend into a less controlled environment? This is an empirical question, and an incredibly valuable research endeavor, both for basic and applied fields. If it is possible to increase water consumption in cats, simply through environmental manipulations, we may be able to reduce the number of cats experiencing kidney failure (which is a common issue in both domestic—Jepson, 2016—and exotic cats, D'Arcy, 2018). For basic research, this provides a clear extension of the laboratory into a more complex environment, which is the definition of translational research (Mace & Critchfield, 2010). This extension illuminates how basic principles translate into more ‘real world’ scenarios. This is simply one of many research endeavors that has clear value for both the basic and applied fields, and one that would not be possible without the skills and knowledge of individuals from both sides.

This brings us back to the difference in scale we started with; the difference between studying an individual animal's behavior in a strictly controlled environment, and studying many individuals in a less controlled environment. This is clearly an issue of scale, and as mentioned earlier, one of the biggest limitations to both the basic research and applied settings when viewed in isolation. Viewing applied practice from solely an applied viewpoint, or basic research from only a basic perspective, is limiting. In order to truly understand behavior, and develop effective practice in the ‘real world’, we need to close the research–practice loop. It is our hope that by viewing basic research and applied practice as extremes on a spectrum, individuals will see the other side as more approachable and accessible. A merging of methods, skills, and approaches requires collaboration with others, as well as expanding our own experiences and points of view. In addition to collaboration among individuals with these different skill sets and experiences, we need individuals that live in both worlds, and can serve as translators and connectors between these two extremes. We need to balance the scale between basic research and applied animal research and practice.

But what does this really mean? How, as individuals, can we begin to work to ‘balance the scale’? The short answer is simply, collaborate. But how? The authors suggest three things. First, work to balance your own scale. We have found that when skill sets overlap, at least a little bit, collaborations are more effective and engaging. Active discussion occurs when there are various points of view and approaches to a given problem/question. This can only happen when people are together that bring both similar passions and different experiences. This should be reflected in not only practice, but in education. With basic laboratories continuing to lose funding (Mace & Critchfield, 2010), and applied programs focusing less and less on basic work, educators should not only be well‐versed in the connections between applied practice and basic research, but work to incorporate these intersections into their teaching and mentoring. Students should not only be encouraged to seek out, but should also be demanding, access to course work and opportunities that will develop the skills and approaches of a basic researcher. As practitioners and researchers, we should all be working to develop multiple areas of strength. This requires a degree of self‐awareness and exploring beyond our areas of comfort.

This brings us to the second recommendation: Broaden your circle. Throughout this piece we have attempted to shed light on how our research questions came to be by highlighting those involved in the conversations that prompted these questions. In these few examples, those people have included veterinarians, shelter staff, animal care staff, students, and (both applied and basic) researchers. These individuals know all too well the struggles and problems they would like solved or questions they would like answered. Talk to them. Volunteer at a shelter or zoo. Be an active listener. If you are an applied animal practitioner and are aware of the questions/struggles in a particular area, then expanding your circle may include individuals whose strengths are different than your own. So often, work in the applied area neglects the findings and appropriate interpretations from the basic literature (if the basic literature is referenced at all). Go to a basic research conference. Read articles from the basic literature. Join or start a basic research discussion group. Bring in a speaker with a vastly different approach from your own for continuing education. Work on finding individuals who have different experiences and backgrounds from your own. Work to become the collaborator that you would want to work with.

Our last suggestion is one that may seem too simplistic to be effective, but has proven to be so time and time again: Reach out to others. Send an email to the author of a paper. Stay after a research talk to ask questions. In our experiences, researchers are hungry for this kind of collaboration. For example, the first iteration of the lick device created for the cat research (mentioned above) used the information outlined by Slotnick, 2009. This device would not have been usable if it had not been for the support and insights from Dr. Slotnick (who provided additional information on a more current design, and even went as far to share an example circuit), and Dr. Dieter Vanderelst, a biologist/engineer/psychologist who graciously offered to collaborate on this project after a cold email was sent by the first author. Of course, reaching out is difficult if you have not yet broadened your circle, or made efforts to ‘balance your own scale' by working to develop your areas of weakness and hone your strengths. It is our hope that if more individuals engage in these behaviors, there will be a greater appreciation for the similarities between the extremes of basic research and applied practice, and our field as a whole will gain a better understanding of behavior, and be better able to apply this knowledge to real‐world problems.
